# A decision tool to help researchers make decisions about including systematic reviews in overviews of reviews of healthcare interventions

**DOI:** 10.1186/s13643-018-0768-8

**Published:** 2019-01-22

**Authors:** Michelle Pollock, Ricardo M. Fernandes, Amanda S. Newton, Shannon D. Scott, Lisa Hartling

**Affiliations:** 1 Alberta Research Centre for Health Evidence, Department of Pediatrics, University of Alberta, 4-472 Edmonton Clinic Health Academy, 11405 87 Avenue NW, Edmonton, AB T6G-1C9 Canada; 2 Clinical Pharmacology Unit, Instituto de Medicina Molecular, University of Lisbon, Lisbon, Portugal; 3 Department of Pediatrics, Santa Maria Hospital, Lisbon, Portugal; 4 Department of Pediatrics, University of Alberta, Edmonton, Canada; 5 Faculty of Nursing, University of Alberta, Edmonton, Canada

**Keywords:** Overview of reviews, Systematic review, Knowledge synthesis, Case series, Decision tool

## Abstract

**Background:**

Overviews of reviews of healthcare interventions (overviews) integrate information from multiple systematic reviews (SRs) to provide a single synthesis of relevant evidence for decision-making. Overviews may identify multiple SRs that examine the same intervention for the same condition and include some, but not all, of the same primary studies. Different researchers use different approaches to manage these “overlapping SRs,” but each approach has advantages and disadvantages. This study aimed to develop an evidence-based decision tool to help researchers make informed inclusion decisions when conducting overviews of healthcare interventions.

**Methods:**

We used a two-stage process to develop the decision tool. First, we conducted a multiple case study to obtain empirical evidence upon which the tool is based. We systematically conducted seven overviews five times each, making five different decisions about which SRs to include in the overviews, for a total of 35 overviews; we then examined the impact of the five inclusion decisions on the overviews’ comprehensiveness and challenges, within and across the seven overview cases. Second, we used a structured, iterative process to transform the evidence obtained from the multiple case study into an empirically based decision tool with accompanying descriptive text.

**Results:**

The resulting decision tool contains four questions: (1) Do Cochrane SRs likely examine all relevant intervention comparisons and available data? (2) Do the Cochrane SRs overlap? (3) Do the non-Cochrane SRs overlap? (4) Are researchers prepared and able to avoid double-counting outcome data from overlapping SRs, by ensuring that each primary study’s outcome data are extracted from overlapping SRs only once? Guidance is provided to help researchers answer each question, and empirical evidence is provided regarding the advantages, disadvantages, and potential trade-offs of the different inclusion decisions.

**Conclusions:**

This evidence-based decision tool is designed to provide researchers with the knowledge and means to make informed inclusion decisions in overviews. The tool can provide practical guidance and support for overview authors by helping them consider questions that could affect the comprehensiveness and complexity of their overviews. We hope this tool will be a useful resource for researchers conducting overviews, and we welcome discussion, testing, and refinement of the proposed tool.

## Background

Overviews of reviews of healthcare interventions integrate information from multiple similar systematic reviews (SRs) to provide a single synthesis of relevant evidence for healthcare decision-making [1]. As the number of published SRs continues to increase [2], it is becoming more common for overview authors to identify multiple relevant SRs that address the same (or very similar) clinical questions and that include many (but not necessarily all) of the same primary studies [3, 4]. Including these “overlapping SRs” in overviews can pose methodological challenges for researchers [3–5]. It can be difficult to appropriately extract and analyze outcome data from overlapping SRs that have variable conduct, quality, and reporting. Further, bias may be introduced into the overview if outcome data from primary studies included in multiple SRs contribute to the analyses more than once.

Different researchers use different approaches to manage overlapping SRs in overviews [3, 4]. Some researchers search for and include all overlapping SRs, but try to extract each primary study’s outcome data only one time, regardless of how many SRs include that primary study [6, 7]. Other researchers attempt to avoid overlap by searching for and including only Cochrane SRs, as Cochrane attempts to avoid duplication by publishing only one SR per topic of interest [1, 8]. Yet other researchers search for all overlapping SRs, but avoid overlap by prioritizing inclusion of only one SR per group of overlapping SRs (e.g., by including only the Cochrane, most recent, or highest quality SR) [6].

In a companion paper by Pollock et al., we examined the impact of the above inclusion decisions on the comprehensiveness and results of overviews, and we documented the challenges associated with these different inclusion decisions [9]. We found that each inclusion decision presented its own unique advantages, disadvantages, and trade-offs, with respect to comprehensiveness of the overviews and challenges encountered. The purpose of the current study was to practically use the above-described study results to develop an evidence-based decision tool to help researchers make informed inclusion decisions when conducting overviews of reviews of healthcare interventions.

## Methods

We used a two-stage process to develop the decision tool. First, we conducted a multiple case study to obtain the empirical evidence upon which the tool is based. We systematically conducted seven overviews five times each, making five different decisions about which SRs to include in the overviews, for a total of 35 overviews. We then examined the impact of the inclusion decisions on the overviews’ comprehensiveness, results, and challenges, both within and across the seven overview cases [9]. Second, we used a structured and iterative process to transform the evidence obtained from the multiple case study into an empirically based decision tool with accompanying descriptive text. Both stages are described in detail below.

### Multiple case study

To develop the decision tool, we first conducted a multiple case study [10]. Each “case” was an overview of healthcare interventions conducted by the Alberta Research Centre for Health Evidence between 2010 and 2016 that examined the efficacy or effectiveness of multiple healthcare interventions for preventing or treating a clinical condition in pediatrics. The seven cases were acute asthma [11], acute otitis media [12], bronchiolitis [13], croup [14], eczema [15], gastroenteritis [16], and procedural sedation [17]. Each of the seven cases were conducted using five different inclusion scenarios (described below), for a total of 35 overviews of healthcare interventions.

For each overview, all published, English-language Cochrane and non-Cochrane SRs that met the overview’s inclusion criteria were identified and included. The seven overviews were then conducted using five different inclusion criteria, for a total of 35 overviews. For the *full inclusion scenario*, all eligible outcome data were extracted from all eligible SRs; accuracy of effect estimates was ensured by extracting each primary study’s outcome data only once, regardless of how many SRs contained that study’s data. For the *first restricted scenario*, all eligible outcome data were extracted from only the Cochrane SRs. For the *last three restricted scenarios*, all eligible outcome data were extracted from all non-overlapping SRs, and for groups of overlapping intervention comparisons within SRs, outcome data were extracted from the following: Cochrane SR (if available), most recent SR (by publication date or search date), or highest quality SR (assessed by two independent reviewers using the AMSTAR tool [18]). Data extraction and analysis for the 35 overviews was conducted by one reviewer using standard methods [19]. For each overview, we extracted descriptive data (for SRs, and their included primary studies) and outcome data (for all relevant outcomes specified in the corresponding overviews). Outcome data were classified using published criteria as “favorable,” “neutral,” “unfavorable,” or “unknown” [9, 20, 21]. We also documented the challenges we encountered related to including overlapping SRs in overviews.

For each overview topic, we described the characteristics of the *full inclusion scenario*. We then systematically compared the *full inclusion scenario* with each of the four *restricted inclusion scenarios* by documenting the number and percentage of SRs, intervention comparisons, primary studies, and subjects that were lost in each restricted scenario and the number and percentage of outcomes that were lost or changed (from favorable, neutral, unfavorable, or unknown, to something else) in each restricted scenario. We then identified and narratively described groups of similar and contrasting overviews [10, 22] and narratively summarized the challenges we encountered when conducting the different inclusion scenarios. The full methods and results are reported in the companion paper by Pollock et al. [9].

### Developing the decision tool

We used a structured and iterative process to transform the results of the multiple case study into an evidence-based decision tool, with accompanying descriptive text, to help researchers make inclusion decisions in overviews. We developed the tool using established principles for designing and populating network displays. This involved reconfiguring the inclusion decisions and study results into an ordered decision model with accompanying descriptive text [22].

In the multiple case study, we found that certain variables differentially affected the comprehensiveness and results of overviews [9]. When creating the decision tool, we transformed those variables into yes/no questions, provided guidance to help researchers answer the questions, and structured the tool so that different responses to the questions corresponded to appropriate inclusion decisions. Thus, the decision tool provides a visual display of the decision points and potential decision pathways available to overview authors. In the multiple case study, we also documented the impact of the inclusion decisions on the comprehensiveness and results of overviews and the challenges we encountered when conducting the different inclusion scenarios [9]. We transformed these results into empirically derived descriptive text explaining the impact, advantages, disadvantages, and potential trade-offs of the different inclusion decisions. Thus, the descriptive text can help authors systematically consider the potential implications of making different inclusion decisions in overviews.

The decision tool was developed iteratively by the same team of researchers who conducted the multiple case study. Thus, all researchers had content and methodological expertise and were familiar with the multiple case study findings. One researcher developed, refined, and finalized the decision tool and its accompanying descriptive text using the above-described process (MP). The additional four researchers oversaw this process and provided several rounds of feedback on the content, placement, and wording of the decision points and pathways in the decision tool, and the content and wording of the descriptive text (RMF, ASN, SDS, LH). All researchers approved the final version of the tool and agreed that it comprehensively reflected the results of the multiple case study. Taken together, the decision tool and its accompanying text can provide overview authors with both the knowledge and the means to make informed inclusion decisions that are specific to the context of their unique overview.

## Results

### Multiple case study results

The seven overviews included in the multiple case study contained 6–19 SRs (range 0–7 Cochrane SRs and 2–13 non-Cochrane SRs). Tables 1 and 2 briefly describe the comprehensiveness and complexity of the different inclusion scenarios; Table 3 is a summary table that compares the different inclusion scenarios. For complete study results, see the companion paper by Pollock et al. [9].

**Table 1:** Comprehensiveness of different inclusion scenarios

Inclusion scenario	Amount of outcome data lost of changed per overview (%)						
	Acute asthma	Acute otitis media	Bronchiolitis	Croup	Eczema	Gastroenteritis	Procedural sedation
Full inclusion scenario (include all Cochrane and non-Cochrane SRs)	0	0	0	0	0	0	0
First restricted scenario (include only Cochrane SRs)	28	54	13	0	67	31	0
Second restricted scenario (include all non-overlapping SRs and select the Cochrane SR for groups of overlapping SRs)	4	39	13	0	39	31	Unknown^a^
Third restricted scenario (include all non-overlapping SRs and select the most recent SR for groups of overlapping SRs)	12	39	13	0	48	34	Unknown^a^
Fourth restricted scenario (include all non-overlapping SRs and select the highest quality SR for groups of overlapping SRs)	4	Unknown^a^	13	0	Unknown^a^	31	Unknown^a^

^a^Unable to calculate amount of outcome data loss and change because data for the comparator groups were often not available (procedural sedation overview) or because multiple systematic reviews were tied for “highest quality” (fourth restricted scenario)

Modified from Pollock et al. [9]

**Table 2:** Complexity of different inclusion scenarios

Inclusion scenario	Description of challenges encountered	Number of overviews affected (out of 7)
Full inclusion scenario (include all Cochrane and non-Cochrane SRs)	Some overlapping primary studies included in non-Cochrane SRs were identified by, but excluded from, the Cochrane SRs for being outside the scope or for having methodological deficiencies.	6
	Overlapping SRs sometimes presented the same or similar outcome data in different ways.	6
	Overlapping SRs sometimes had discordant results for the same outcomes.	5
	Data extraction from non-Cochrane SRs was sometimes difficult due to deficiencies in conduct and reporting.	6
First restricted scenario (include only Cochrane SRs)	Input from a clinical expert was often required to determine whether the Cochrane SRs comprehensively examined all relevant intervention comparisons.	6
	Cochrane SRs sometimes overlapped.	2
Second restricted scenario (include all non-overlapping SRs and selects the Cochrane SR for groups of overlapping SRs)	Not all groups of overlapping SRs included a Cochrane SR.	2
	Data extraction from non-Cochrane SRs was sometimes difficult due to deficiencies in conduct and reporting.	6
Third restricted scenario (include all non-overlapping SRs and select the most recent SR for groups of overlapping SRs)	Overlapping SRs were sometimes “tied” for most recent year of publication.	3
	Search dates were not comprehensively or consistently reported in all SRs.	6
	Data extraction from non-Cochrane SRs was sometimes difficult due to deficiencies in conduct and reporting.	6
Fourth restricted scenario (include all non-overlapping SRs and select the highest quality SR for groups of overlapping SRs)	Overlapping SRs were sometimes “tied” for highest quality.	3
	Conducting quality assessments was challenging and time-intensive.	7
	Data extraction from non-Cochrane SRs was sometimes difficult due to deficiencies in conduct and reporting.	6

Modified from Pollock et al. [9]

**Table 3:** Summary of different inclusion scenarios on comprehensiveness and complexity of overviews

Inclusion scenario	Impact on comprehensiveness of overviews, compared to other inclusion scenarios	Impact on complexity of overviews, compared to other inclusion scenarios
Full inclusion scenario (include all Cochrane and non-Cochrane SRs)	Most comprehensive	Most challenging
First restricted scenario (include only Cochrane SRs)	Least comprehensive	Least challenging
Second restricted scenario (include all non-overlapping SRs and select the Cochrane SR for groups of overlapping SRs)	Less comprehensive than full inclusion scenario, but often more comprehensive than first restricted scenario	Less challenging than full inclusion scenario, but often more challenging than first restricted scenario
Third restricted scenario (include all non-overlapping SRs and select the most recent SR for groups of overlapping SRs)	Less comprehensive than second restricted scenario	More challenging than second restricted scenario
Fourth restricted scenario (include all non-overlapping SRs and select the highest quality SR for groups of overlapping SRs)	Same as second restricted scenario	More challenging than second restricted scenario

### Decision tool to help researchers make inclusion decisions in overviews

**Fig. 1:**
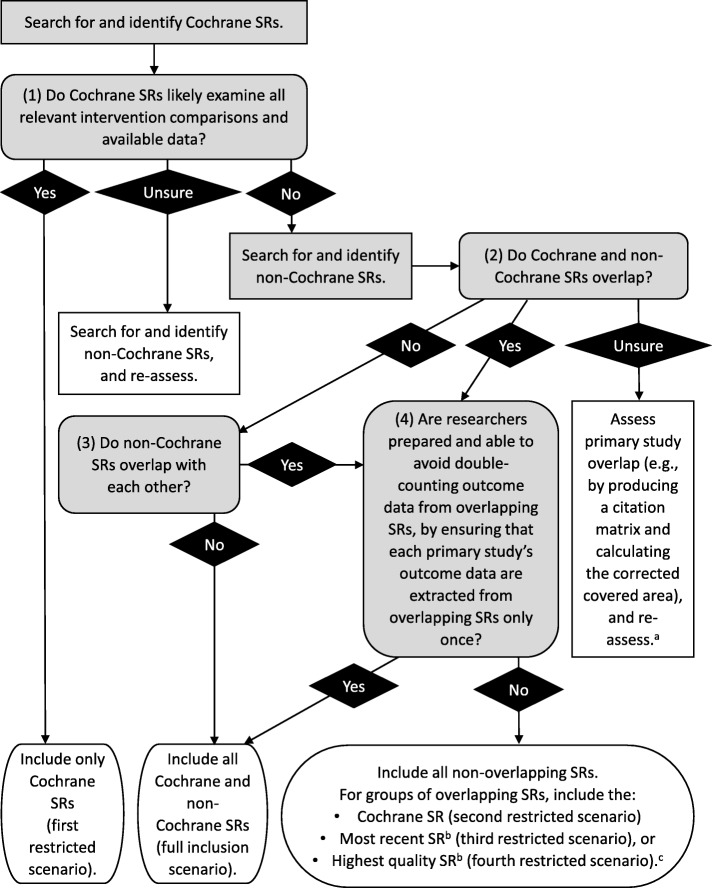
Decision tool to help researchers make inclusion decisions in overviews. ^a^For detailed instructions on assessing primary study overlap, see Pieper et al. [5]; ^b^Researchers should clearly operationalize the criteria they use to define “most recent” and “highest quality.” ^c^For groups of overlapping SRs, researchers may choose to include the most relevant SRs or the most comprehensive SRs (though these inclusion decisions were not examined in this methods study)

The above-described results were used to develop an evidence-based decision tool, with empirically derived accompanying text, to provide practical guidance for researchers making inclusion decisions in overviews. The decision tool is presented in Fig. 1. The accompanying descriptive text regarding the impact, advantages, disadvantages, and potential trade-offs of the different inclusion decisions is presented below.

#### Decision point 1: do Cochrane SRs likely examine all relevant intervention comparisons and available data?

If yes, researchers may choose to include only Cochrane SRs in the overview. An advantage (and potential disadvantage) of this inclusion decision is that researchers are likely (but not guaranteed) to avoid issues related to overlapping SRs (described below in decision point 4). A disadvantage (and potential advantage) is that there will likely (but not always) be some data loss from non-Cochrane SRs for overlapping intervention comparisons; however, it is unclear whether these additional data are of clinical importance, since some data may come from primary studies in non-Cochrane SRs that were identified by, but excluded from, the Cochrane SRs, for being outside their scope or having methodological deficiencies. Input from clinical experts and other stakeholders may be required to assess whether the Cochrane SRs comprehensively examine all relevant intervention comparisons and available data. Cochrane SRs may be deemed not comprehensive if they do not examine all relevant intervention comparisons or contain all available primary studies (e.g., if they are not relatively current or up to date). Researchers who are not comfortable answering this question based on the Cochrane SRs alone may opt to search for and identify non-Cochrane SRs [3] and re-assess. In cases where Cochrane SRs overlap, authors may either include all overlapping Cochrane SRs and take steps to avoid double-counting outcome data or include only the most recent or highest quality Cochrane SR. Both of these options are described in more detail below (see decision point 4).

#### Decision points 2 and 3: do the included SRs overlap?

If researchers suspect that the Cochrane SRs are not comprehensive, the next decision point asks them to search for and identify non-Cochrane SRs and determine whether the primary studies contained within the included SRs overlap. Researchers may wish to examine the primary study overlap separately for each set of intervention comparisons, as SRs often contain multiple comparisons. If, across all intervention comparisons, the Cochrane and non-Cochrane SRs do not overlap, and the non-Cochrane SRs do not overlap with each other, researchers can include all Cochrane and non-Cochrane SRs in the overview without concern for issues related to double-counting outcome data. This situation was not observed in the current methods study and is likely to be rare. If researchers are unsure whether or to what extent the SRs overlap, they can assess overlap by producing a citation matrix and using it to calculate the corrected covered area [5].

#### Decision point 4: are researchers prepared and able to avoid double-counting outcome data from overlapping SRs, by ensuring that each primary study’s outcome data are extracted from overlapping SRs only once?

When the included SRs overlap, researchers may opt to include all Cochrane and non-Cochrane SRs if they are prepared and able to avoid challenges related to including overlapping SRs and extracting and analyzing their outcome data. An advantage of this inclusion decision is that it is the only way to ensure that all data from all SRs are included in the overview. A disadvantage is that researchers are likely to encounter multiple challenges when including overlapping SRs in overviews. As described above (decision point 1), primary studies contained within non-Cochrane SRs may have been identified by (but excluded from) the Cochrane SRs for reasons related to eligibility criteria or methodological quality, making it difficult to determine whether and which data from non-Cochrane SRs are clinically relevant. When extracting outcome data, overlapping SRs may analyze and present the same or similar outcome data in different ways and may have discordant results for the same outcome. Further, overlapping, non-Cochrane SRs may be older and of lower quality and may have deficiencies in their conduct and reporting that makes data extraction difficult. All of these challenges may make it difficult for researchers to extract outcome data from overlapping SRs in a systematic and transparent way and may also increase the complexity of other stages of the overview process.

If researchers cannot avoid double-counting outcome data from overlapping SRs, they may opt to balance comprehensiveness and complexity by including all non-overlapping SRs; for groups of overlapping SRs, they may opt to include the Cochrane, most recent, or highest quality SR. Using specific criteria to prioritize SR inclusion when confronted with multiple, overlapping SRs can allow researchers to capitalize on the advantages of the previous two inclusion scenarios by avoiding potential issues related to double-counting outcome data while maximizing the amount of data included in the overview. For groups of overlapping SRs, including Cochrane SRs compared to the most recent or highest quality SRs may most effectively minimize both data loss and methodological issues. Researchers may face the following challenges if including the most recent or highest quality SRs for groups of overlapping SRs: recency of SRs may be assessed and operationalized in different ways, some SRs may be “tied” for most recent or highest quality, and conducting quality assessments for the purpose of selecting eligible SRs may be challenging and time-intensive. However, if including the Cochrane SRs for groups of overlapping SRs, researchers should be aware that multiple Cochrane SRs may contribute outcome data to the same comparison (i.e., Cochrane SRs may sometimes overlap), and not all groups of overlapping SRs may include a Cochrane SR.

## Discussion

In a multiple case study of seven overviews conducted using five different inclusion decisions, we found that different inclusion decisions differentially affected the comprehensiveness and results of overviews and were associated with different types of challenges [9]. We used these findings to systematically develop an evidence-based decision tool to help researchers make informed inclusion decisions in overviews. Using an evidence-based tool to make inclusion decisions can help promote transparency and rigor and decrease bias. We hope this tool will be a useful resource for researchers conducting overviews, and we welcome discussion, testing, and refinement of the proposed tool.

There are practical considerations involved when using the decision tool in overviews. Specifically, four conditions should be met prior to its use. First, the overview should examine multiple interventions for preventing or treating a health condition, though further testing and real-life application of the tool will help determine whether it can be used with other types of overviews [1]. Second, the overview format should be more appropriate than the SR format to answer the research question [3]. Third, researchers should intend to search for and include only SRs in the overview [1, 3]. Fourth, researchers should be prepared and able to avoid double-counting outcome data from overlapping SRs, either by not including overlapping SRs in the overview or by ensuring that each primary study’s outcome data are extracted from overlapping SRs only once [3–5]. The layout of the tool is based on two additional considerations. First, we acknowledge that researchers may decide to prioritize Cochrane SRs for inclusion in overviews due to both their higher methodological rigor (on average) [2] and the additional time, skills, resources, and challenges associated with searching for, including, and extracting data from non-Cochrane SRs [1, 3]. The tool’s layout ensures that researchers consider the potential implications of this inclusion decision upfront, in the context of their specific overview question. Second, we anticipate that some questions in the decision tool may be difficult to answer. To address this, we identified two points in the tool where researchers may wish to gather more information to help inform their decision.

This decision tool can provide practical guidance and support for overview authors by helping them make transparent and informed inclusion decisions when faced with overlapping SRs in overviews. As the number of overlapping (and possibly conflicting) SRs continues to increase [23], issues surrounding the inclusion of overlapping SRs in overviews will likely become more complex. This decision tool may help address this complexity. It can help authors consider questions that can affect the nature and extent of outcome data included and not included in overviews, as well as the impact, advantages, disadvantages, and potential trade-offs of making different inclusion decisions in overviews. The decision tool may also help researchers structure the decision-making process and appropriately contextualize their overview findings in light of the potential completeness of the overview’s evidence base. Though we did not examine the impact of the different inclusion decisions on overview quality, as there is currently no accepted tool to assess quality of overviews, we hope that using the decision tool can increase the transparency and reproducibility of the methods used and decisions made. Importantly, the decision tool is not designed to tell researchers what to do. Instead, it is designed to provide researchers with both the knowledge and the means to make their own informed inclusion decisions. As such, each author team must consider the unique characteristics of their overview, including its purpose, scope, target audience, and resource availability, when determining the most appropriate course of action.

The inclusion decisions presented in the decision tool and examined in the companion paper by Pollock et al. [9] are described in the literature as common ways to manage overlapping SRs in overviews while avoiding issues related to double-counting primary studies’ outcome data [3, 4, 8]. However, researchers may sometimes make other inclusion decisions motivated by reasons other than considerations about overlapping SRs and double-counting data. For example, researchers that suspect that Cochrane SRs are comprehensive may still opt to search for and potentially include non-Cochrane SRs in the overview, while those that suspect that Cochrane SRs are not comprehensive may opt to include only Cochrane SRs and discuss this as a study limitation. Other researchers who are unable to avoid double-counting outcome data may still opt to include all Cochrane and non-Cochrane SRs and discuss this as a study limitation. Yet other researchers may use results of quality assessments as an exclusion criterion. They may also manage groups of overlapping SRs by choosing to include the “most comprehensive SRs” or the “most relevant SRs” (though these subjective assessments may be operationalized in different ways depending on the overview topic and thus were not examined in this methods study). Though outside the scope of the current study, research investigating the implications of these other inclusion decisions in overviews is needed. Further, the seven overviews used in the companion paper by Pollock et al. [9] all examined interventions in pediatric health, and future research and practical application of the decision tool is also needed to help determine the utility of the tool in a broader sample of overviews of healthcare interventions. Lastly, though we developed the decision tool for use in overviews, future research and practical application of the tool may also find it to be useful for researchers conducting other knowledge syntheses that include SRs.

## Conclusions

Different researchers currently make different inclusion decisions in overviews, but may not understand the ways in which these inclusion decisions affect the comprehensiveness and complexity of their overviews. This study used empirical findings to develop an evidence-based decision tool to help researchers make transparent and informed inclusion decisions in overviews. The tool can provide practical guidance and support for overview authors in two ways: by helping them consider questions that can affect the comprehensiveness and complexity of their overviews and by helping them assess the potential impact, advantages, disadvantages, and trade-offs of the different inclusion decisions. The tool may be useful when developing overview protocols and/or when conducting and reporting overviews. This tool would benefit from additional discussion, testing, and refinement. It may also have relevance to other knowledge synthesis products that include SRs.

## Abbreviations

AMSTAR: A MeaSurement Tool to Assess systematic Reviews
Overview: Overview of reviews
SR: Systematic review

## References

[1] Becker LA, Oxman AD, JPT H, Green S (2011). Chapter 22: overviews of reviews. Cochrane handbook for systematic reviews of interventions (version 5.1.0). The Cochrane Collaboration.

[2] Page MJ, Shamseer L, Altman DG, Tetzlaff J, Sampson M, Tricco AC (2016). Epidemiology and reporting characteristics of systematic reviews of biomedical research: a cross-sectional study. PLoS Med.

[3] Pollock M, Fernandes RM, Becker LA, Featherstone R, Hartling L (2016). What guidance is available for researchers conducting overviews of reviews of healthcare interventions? A scoping review and qualitative metasummary. Syst Rev.

[4] Ballard M, Montgomery P (2017). Risk of bias in overviews of reviews: a scoping review of methodological guidance and four-item checklist. Res Synth Methods.

[5] Pieper D, Antoine SL, Mathes T, Neugebauer EA, Eikermann M (2014). Systematic review finds overlapping reviews were not mentioned in every other overview. J Clin Epidemiol.

[6] Cooper H, Koenka AC (2012). The overview of reviews: unique challenges and opportunities when research syntheses are the principal elements of new integrative scholarship. Am Psychol.

[7] Caird J, Sutcliffe K, Kwan I, Dickson K, Thomas J (2015). Mediating policy-relevant evidence at speed: are systematic reviews of systematic reviews a useful approach?. Evid Policy.

[8] Thomson D, Foisy M, Oleszczuk M, Wingert A, Chisholm A, Hartling L (2013). Overview of reviews in child health: evidence synthesis and the knowledge base for a specific population. Evid Based Child Health..

[9] Pollock M, Fernandes RM, Newton AS, Scott SD, Hartling L. The impact of different inclusion decisions on the comprehensiveness and complexity of overviews of reviews of healthcare interventions. Syst Rev. 2019;8:18. 10.1186/s13643-018-0914-3. 10.1186/s13643-018-0914-3 PMC6329144 30635048

[10] Yin RK (2013). Case study research: design and methods.

[11] Pollock M, Sinha I, Hartling L, Rowe BH, Schrieber S, Fernandes RM (2017). Inhaled short-acting bronchodilators for managing emergency childhood asthma: an overview of reviews. Allergy.

[12] Oleszczuk M, Fernandes RM, Thomson D, Shaikh N (2012). The Cochrane Library and acute otitis media in children: an overview of reviews. Evid Based Child Health.

[13] Bialy L, Foisy M, Smith M, Fernandes RM (2011). The Cochrane Library and the treatment of bronchiolitis in children: an overview of reviews. Evid Based Child Health.

[14] Bjornson C, Russell K, Foisy M, Johnson DW (2010). The Cochrane Library and the treatment of croup in children: an overview of reviews. Evid Based Child Health.

[15] Foisy M, Boyle RJ, Chalmers JR, Simpson EL, Williams HC (2011). The prevention of eczema in infants and children: an overview of Cochrane and non-Cochrane reviews. Evid Based Child Health.

[16] Freedman SP, Ali S, Oleszczuk M, Gouin S, Hartling L (2013). Treatment of acute gastroenteritis in children: an overview of systematic reviews of interventions commonly used in developed countries. Evid Based Child Health.

[17] Hartling L, Milne A, Foisy M, Lang E, Sinclair D, Klassen TP (2016). What works and what’s safe in pediatric emergency procedural sedation: an overview of reviews. Acad Emerg Med.

[18] Shea BJ, Grimshaw JM, Wells GA, Boers M, Andersson N, Hamel C (2007). Development of AMSTAR: a measurement tool to assess the methodological quality of systematic reviews. BMC Med Res Methodol.

[19] Deeks JJ, HJP T, Altman DG, JPT H, Green S (2011). Chapter 9: analysing data and undertaking meta-analyses. Cochrane handbook for systematic reviews of interventions (version 5.1.0). The Cochrane Collaboration.

[20] Tricco AC, Tetzlaff J, Pham B, Brehaut J, Moher D (2009). Non-Cochrane vs. Cochrane reviews were twice as likely to have positive conclusion statements: cross-sectional study. J Clin Epidemiol.

[21] Lai NM, Teng CL, Lee ML (2011). Interpreting systematic reviews: are we ready to make our own conclusions? A cross-sectional study. BMC Med.

[22] Miles MB, Huberman AM, Saldana J (2014). Qualitative data analysis: a methods sourcebook.

[23] Ioannidis JP (2016). The mass production of redundant, misleading, and conflicted systematic reviews and meta-analyses. Milbank Q.

